# Emergency Contraception Access and Counseling in Urban Pharmacies: A Comparison between States with and without Pharmacist Prescribing

**DOI:** 10.3390/pharmacy8020105

**Published:** 2020-06-19

**Authors:** Rebecca H. Stone, Sally Rafie, Dennia Ernest, Brielle Scutt

**Affiliations:** 1 College of Pharmacy, University of Georgia, Athens, GA 30605, USA; 2 Birth Control Pharmacist, San Diego, CA 92122, USA; sally@birthcontrolpharmacist.com; 3 Department of Pharmacy, UC San Diego Health, San Diego, CA 92103, USA; 4 Department of Pharmacy, West Texas VA Health Care System, Big Spring, TX 79720, USA; dennia.Ernest@va.gov; 5 Department of Pharmacy, Johns Hopkins Hospital, Baltimore, MD 21231, USA; bscutt2@jhmi.edu

**Keywords:** emergency contraception, levonorgestrel, ulipristal, contraception, hormonal contraception, counseling, medication access, pharmacist prescribing, pharmacist scope

## Abstract

Pharmacists are often the primary source of emergency contraception (EC) access and patient information. This study aims to identify differences in pharmacist-reported EC access and counseling between states which do or do not permit pharmacist-prescribed EC. This prospective, mystery caller study was completed in California (CA), which permits pharmacist-prescribed EC after completion of continuing education, and Georgia (GA), which does not. All community pharmacies that were open to the public in San Diego and San Francisco, CA, and Atlanta, GA were called by researchers who posed as adult females inquiring about EC via a structured script. Primary endpoints were EC availability and counseling. Statistical analyses completed with SPSS. Researchers called 395 pharmacies, 98.2% were reached and included. Regarding levonorgestrel (LNG), CA pharmacists more frequently discussed (CA 90.4% vs. GA 81.2%, *p* = 0.02), stocked (CA 89.5% vs. GA 67.8%, *p* < 0.01), and correctly indicated it “will work” or “will work but may be less effective” 4 days after intercourse (CA 67.5% vs. GA 17.5%, *p* < 0.01). Ulipristal was infrequently discussed (CA 22.6% vs. GA 3.4%, *p* < 0.01) and rarely stocked (CA 9.6% vs. GA 0.7%, *p* < 0.01). Pharmacists practicing in states which permit pharmacist-prescribed EC with completion of required continuing education may be associated with improved patient access to oral EC and more accurate patient counseling.

## 1. Introduction

Currently there are three types of emergency contraception (EC) available in the United States: insertion of the copper intrauterine device (Cu-IUD), an over the counter levonorgestrel (LNG) 1.5 mg oral tablet, and a prescription-only ulipristal acetate (UPA) 30 mg oral tablet [1]. 

The Cu-IUD is used off-label for EC and is the most effective method available; however, compared to oral EC methods, it is infrequently used, likely due to issues surrounding timely access and lack of patient and provider awareness [2]. Oral LNG was the first dedicated progestin-only EC pill, and was approved by the FDA in 1999 as a two 0.75 mg dose regimen and required a prescription [3]. Over time, it was formulated as a single 1.5 mg dose regimen and approved over the counter for individuals 18 or older in 2006, then 17 and older in 2009, before eventually becoming available over the counter to people of all ages in 2013. Ulipristal acetate was FDA-approved as a prescription-only medication in 2010. Ulipristal acetate is more effective than LNG at preventing pregnancy in some situations, particularly in patients with a body weight over 70 kg or 155 pounds, body mass index over 25 kg/m^2^, or when used 4 to 5 days after intercourse [1,4,5,6]. 

In order for a patient to obtain an EC method that best meets their individual needs and situation, they must have timely access to accurate information and the product. Pharmacies are the primary source for obtaining EC, and pharmacists are often the sole source of EC information and counseling for patients, particularly since LNG became available over the counter [3]. However, multiple studies have demonstrated that women frequently encounter barriers to access: both prescribers and pharmacists may have knowledge deficits about EC in general [7,8], and are unfamiliar with or unaware of the more effective oral product, UPA [9]. Additionally, the vast majority of pharmacies do not have UPA readily available, and a small percentage may not have any form of oral EC available [10,11,12,13]. 

One strategy used to improve knowledge and use of EC has been to expand access to both prescription and non-prescription products in pharmacies [3,14]. Although LNG is now available over the counter for people of all ages, EC prescriptive authority for pharmacists remains relevant for insurance coverage and for access to UPA [15]. Starting with California in 2004, there are currently seven states which allow pharmacists to prescribe EC [3]. Most states require pharmacists to complete continuing education specific to EC in order to use this expanded scope of practice. Initially, pharmacist EC prescribing was designed to facilitate LNG access because it was the only dedicated EC product available in the United States until 2010. The language of these protocols often indicate pharmacists are able to prescribe “oral emergency contraception”, but then specifically list LNG in their protocol or reporting documentation, which subsequently prevents or confuses pharmacist prescribing of UPA if the protocol was not amended to include UPA after it was brought to market [3]. 

Finally, in the last 5 years, pharmacist involvement in providing reproductive health services has continued to expand, and a growing number of states have implemented or are in the process of developing legislation which allows pharmacist prescribing of other types of hormonal contraceptives, in addition to EC [3,16]. States which offer pharmacist prescribing of these products require that pharmacists complete additional education and training on these topics in order to prescribe them. However, despite the growing national trend to expand community pharmacist involvement in prescribing contraceptives, there are many states, including Georgia, which still do not permit prescribing of EC or any other type of hormonal contraceptives. 

Although there are multiple mystery shopper studies which evaluate availability and counseling points provided with EC, to our knowledge there is no published data directly comparing EC access and pharmacist counseling between states that allow pharmacists to prescribe EC and those that do not. The objective of this study is to determine if there are differences in patient EC access and counseling between pharmacists located in a state allowing pharmacist prescribing of EC versus a state without this expanded scope of practice. The secondary objective is to determine if there are further differences in EC access and counseling by pharmacy type within each state. 

## 2. Materials and Methods 

**Figure 1:**
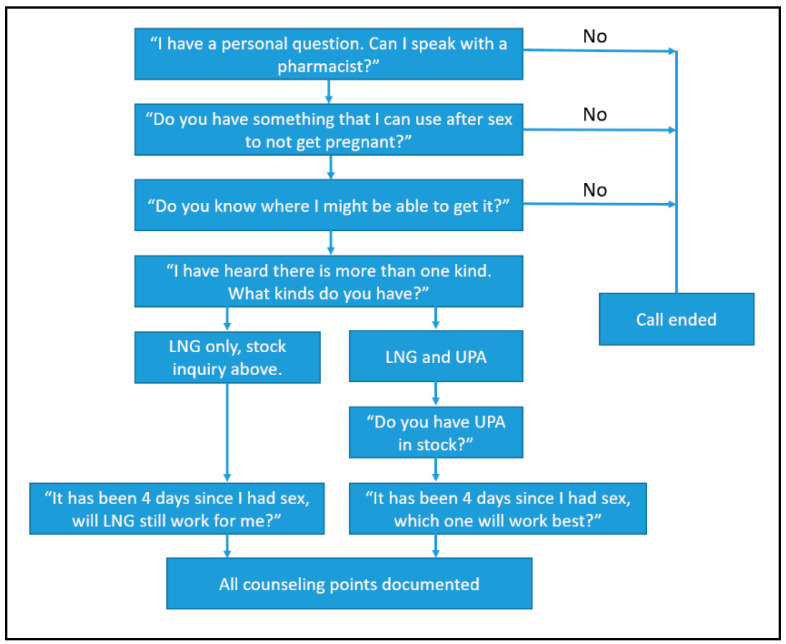
Mystery Caller Telephone Script.

This is a prospective, telephone-based mystery caller survey. Pharmacies located in the cities of Atlanta, Georgia, and San Diego and San Francisco, California, were identified through a list of licensed retail pharmacies obtained from each respective state board of pharmacy. The lists were then filtered to include only retail pharmacies with active licenses that were open to the public. These three cities have comparable levels of urbanization according to National Center for Health Statistics Urban-Rural classification scheme, falling into the most urban categorization of “large central metro,” and were selected as a convenience sample [17]. Three investigators completed calls between November 2016 and July 2017. The investigators developed a structured script (Figure 1), and research students called posing as young adult females requesting to speak to a pharmacist and inquire about pregnancy prevention options after having unprotected sex. The student research callers were trained by the two primary investigators in each state, and practice calls were completed until each research student demonstrated appropriate and consistent delivery of the mystery caller script. Any questions regarding calls were triaged to the two primary investigators. The primary investigators created a standardized data collection form which was used by all the student research callers. The primary endpoints were initial EC method discussed by the pharmacist, reported EC availability, and prompted EC counseling points. Prompted EC counseling points included asking if there is more than one kind of EC product available and if EC works 4 days after unprotected sex. Unprompted pharmacist counseling points that were recorded included discussion regarding the EC window of efficacy, when unprotected intercourse occurred, age, body weight, sexually transmitted infection counseling, ongoing contraception, insurance coverage, and if the patient has a prescription for EC.

Calls were completed on Monday through Saturday between the hours of 8:00 a.m. and 8:00 p.m. If a pharmacist was unavailable or the phone was not answered, the investigator made two additional call attempts. The call was determined completed if the investigator was able to speak with a pharmacist. Pharmacies were identified as independent or chain if they had four or more locations [18], including drugstores (CVS, Walgreens, etc.), grocery stores (Albertsons, Kroger, etc.), and mass merchants (Walmart, Costco, etc.). 

Categorical variables were characterized with descriptive statistics and analyzed with chi-square. Statistical analysis was completed using IMB SPSS Statistics 25. The University of Georgia and University of California San Diego Human Research Protections Program institutional review boards approved the study protocol. 

## 3. Results

Researchers called 395 pharmacies that had an active retail license and were open to the public in these three cities; seven pharmacists were not available or refused to take the call, and were not included in data analysis. Data were collected and analyzed from conversations with the remaining 388 pharmacists (98.2%) (CA 239, 98.4% vs. GA 149, 98.0%). Phone calls took between 30 s and 25 min. Approximately one-third of the pharmacies included were in Georgia (Atlanta 149, 38.4%) and two-thirds in California (CA 239, 61.6%; San Diego 127, 32.7%; San Francisco 112, 28.9%). The majority were chain pharmacies (310, 79.9%), which included drugstore pharmacies, (216, 55.7%), grocery store pharmacies (68, 17.5%), and mass merchandisers (26, 6.7%) (Table 1). Independent pharmacies composed 20.1%, and there were no differences between states when evaluating the proportion of independent pharmacies versus chain pharmacies (chain pharmacies: CA 42, 17.6% vs. GA 36, 24.2%, *p* = 0.12.)

**Figure 2:**
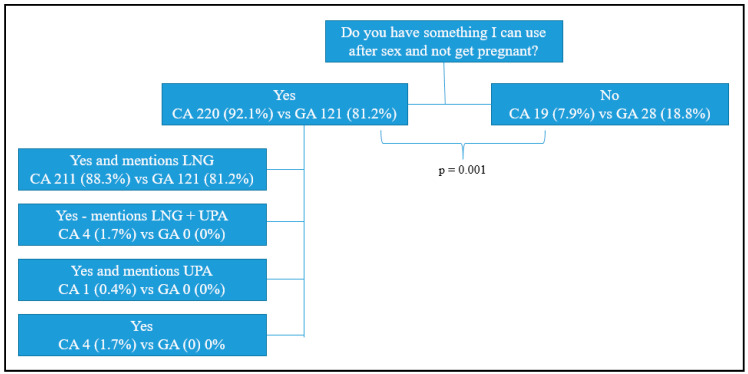
Pharmacist initial response to mystery caller emergency contraception (EC) inquiry.

After the mystery caller patient inquired about available options for pregnancy prevention following unprotected sex, most pharmacists indicated that EC is available (CA 220, 92.1% vs. GA 121, 81.2%, *p* < 0.01), see Figure 2. The majority initially presented LNG alone (CA 211, 88.3% vs. GA 121, 81.2%), while a very small percentage of California pharmacists mentioned both LNG and UPA (4, 1.7%) or UPA alone (1, 0.4%). Four (1.7%) California pharmacists indicated EC was available but did not mention an EC method by name, and the remainder did not offer an EC method (CA 19, 7.9% vs. GA 28, 18.8%). 

Levonorgestrel was discussed by most pharmacists during the call (CA 216, 90.4% vs. GA 121, 81.2%, *p* = 0.01) (Table 2). A majority of pharmacists in both states reported LNG was currently in stock; however, a significantly larger percentage of California pharmacists reported it stocked compared to Georgia pharmacists (CA 214, 89.5% vs. GA 101, 67.8%, *p* < 0.01). Of the pharmacists who reported LNG was in stock, slightly more than half had it available on the OTC shelf and there was no difference in OTC stocking practices between states (Total 184, 58.4%; CA 122, 57.0% vs. GA 62, 61.4%, *p* = 0.46). When comparing chain versus independent pharmacies, independent pharmacies in both states were less likely to discuss or have LNG available in stock (Table 3). 

Lastly, pharmacists in California were more likely to correctly indicate that LNG “will work” or “will work but may be less effective” when used 4 days after intercourse than those in Georgia (CA 112, 67.5% vs. GA 21, 17.5%, *p* < 0.01) (Table 4). 

Pharmacists in both states discussed UPA much less frequently than LNG, but the frequency was particularly low in Georgia (CA 54, 22.6% vs. GA 5, 3.4%, *p* < 0.01) (Table 2). In response to the caller’s initial inquiry, there were no pharmacists in Georgia who presented UPA, and only 9.3% in California who presented it. If pharmacists initially presented LNG alone, then the mystery caller asked additional probing questions, specifically, “I heard there’s more than one kind. What kinds do you have or recommend?” and mentioned that unprotected sex had occurred four days prior. The majority of pharmacists who identified UPA did so after this prompting (CA 49, 20.5% vs. GA 5, 3.4%). Furthermore, only a minority of pharmacists who discussed UPA during the call had it in stock (CA 23, 42.6% vs. GA 1, 20%, *p* = 0.33). When comparing chain versus independent pharmacies there were no statistically significant differences in the frequency of pharmacist discussion or stock availability of UPA (Table 3). 

Of the pharmacists who discussed UPA, when asked “it has been 4 days since I had sex, which one will work best,” most identified that UPA is more effective than LNG (CA 44, 81.5% vs. GA 5, 100%). Of the remaining California pharmacists who responded to the prompt, 5 (9.3%) indicated LNG and UPA were equally effective, 1 (1.9%) indicated they did not know, 1 (1.9%) indicated the copper IUD was more effective than oral methods, and 3 (5.6%) did not respond. 

Unprompted pharmacist counseling points were documented during the call, and there were no differences between states in addressing the time frame of EC efficacy, patient body weight, sexually transmitted infection prevention, ongoing contraception, or patient insurance (Table 5). However, Georgia pharmacists were more likely to unnecessarily inquire about the patients age (CA 5, 2.3% vs. GA 8, 6.6%, *p* = 0.05), and less likely to inquire about a prescription (CA 38, 17.6% vs. GA 0, 0%, *p* < 0.01) and when unprotected intercourse occurred (CA 45, 20.8% vs. GA 14, 11.6%, *p* = 0.03. 

## 4. Discussion

Pharmacists in states which permit them to prescribe EC may be more likely to provide patients with same day oral EC access, which could potentially improve time to administration and the subsequent efficacy of these products. Other studies conducted since LNG became available over the counter have found that it is available for same day purchase in at least 80% of the pharmacies evaluated nationwide [11,19,20,21]. In our study, approximately 90% of California pharmacists reported that LNG was in stock, while Georgia pharmacies were well below the national average, at only 68%. This study found that only about 60% of pharmacies in both states had this product out on the OTC aisle, which is similar to data from other studies [19]. Despite the removal of age restrictions and transition to over the counter status in 2013, studies have found that stores frequently cite theft prevention as reason to store this medication behind the counter. 

When analyzing by pharmacy type, independent pharmacies were less likely than chain pharmacies to discuss or stock LNG. This may be because chain pharmacies are able to maintain a larger formulary of stock on hand for customer convenience, and because they may have internal processes designed to help ensure this product is available. Additionally, independent pharmacies may employ a larger percentage of pharmacists who do not want to dispense EC for personal reasons, and unlike a chain, an independent pharmacy owner may autonomously decide what types of medications the pharmacy will provide. 

Additionally, studies have demonstrated that UPA is rarely stocked in pharmacies across the country, with between 3% and 10% of pharmacies reporting the product in stock [10,12,21,22]. Again, data from our study approached both ends of this range, with California pharmacies at the high end of the national average at 9.6%, and Georgia pharmacies below the national average, where less than 1% of pharmacists reported that UPA was in stock. This is likely related to the fact that many pharmacists and other prescribers are unaware of UPA, with one national study indicating that slightly less than 30% of reproductive health prescribers had heard of UPA, and only 7% had ever prescribed it [9]. There were no differences between chain and independent pharmacies in discussing or stocking UPA, likely in part because so few pharmacists identified it overall. 

Pharmacies generally do not stock medications that are infrequently prescribed due to the shelf life and potential expiration of inventory. Although the cost of UPA to the pharmacy may vary between specific wholesalers, the cost of UPA for patients without insurance is approximately $50 [23]. Therefore, it can be assumed that pharmacies would lose potentially $50 or less if the product expired before use. Perhaps pharmacists who can prescribe UPA are more likely to keep it stocked since they may have an opportunity to initiate the prescription themselves. For example, they may be more likely to recommend and provide this more effective product to patients who are seeking OTC LNG, unlike pharmacists who are dependent upon a patient bringing in a prescription for a medication that is not well known by prescribers.

Perhaps most importantly, pharmacists who have more training in provision of EC are likely to have additional knowledge and counseling skills in this area. This is important because pharmacists are often the only medical professional that patients encounter when seeking EC, particularly since LNG became available OTC. Since pharmacists are the front line for EC, they often have an opportunity to provide patient education and aid in product selection. In this study, when a mystery caller inquired about EC the vast majority of pharmacists initially presented LNG. Data indicates that UPA is likely more effective than LNG, particularly when used 72–120 h after intercourse or for women weighing more than 155 pounds [4]. However, only a small percentage of California pharmacists initially presented UPA or both options to the caller, none of the Georgia pharmacists initially discussed UPA. Only about 22% of California pharmacists ultimately discussed UPA at any point during the call, compared to less than 5% of Georgia pharmacists. As mentioned previously, awareness of UPA is low amongst both pharmacists and other prescribers [9], and the disparity between states in pharmacist discussion of UPA is likely related to pharmacist exposure to this product through the additional curriculum, continuing education and training courses required to prescribe EC. 

When assessing accuracy of pharmacist counseling regarding oral EC, there were also significant differences between prescribing and non-prescribing states. The package insert for LNG indicates it should be used within 72 h of unprotected intercourse; however, it is well known and endorsed by groups such as the American College of Obstetrician and Gynecologists (ACOG) and the US Selected Practice Recommendations for Contraceptive Use, that LNG may work, although it may be less effective, for up to 120 h [1,24]. Over two-thirds of California pharmacists correctly indicated that LNG “will work” or “will work but may be less effective”, compared to just 18% of Georgia pharmacists. One potential explanation for this significant difference is that California pharmacists who prescribe EC are introduced to the LNG 120 h window of efficacy through guideline driven continuing education, while Georgia pharmacists may be more likely to refer to the less comprehensive package insert. However, of the pharmacists who did discuss UPA, the majority in California and all five in Georgia identified that it is more effective than LNG when asked to compare the two. The authors postulate that if pharmacists are aware of UPA, which is relatively unknown to the vast majority of pharmacists and providers [9,10], then they have likely received specific education or training for all oral EC products, including UPA efficacy. 

When considering other unprompted counseling points, Georgia pharmacists were more likely to inquire about patient age, which is no longer necessary since there are no age restrictions for OTC purchases, and may discourage some patients from purchasing this medication. California pharmacists were more likely to ask how long it had been since unprotected intercourse or if the patient had a prescription, which may be related to a larger percentage of pharmacists who are considering prescription-only UPA, because it maintains its efficacy longer and the pharmacist has the ability to write the prescription for the patient if needed. There was virtually no counseling offered by pharmacists in either state regarding unprompted points such as patient body weight, sexually transmitted infection prevention, ongoing contraception, or patient insurance. This may represent a missed opportunity for the pharmacist to offer additional pertinent reproductive health information or refer the patient to more comprehensive care. One small study demonstrated that a 5 to 10 min EC counseling session with a student pharmacist improved patient knowledge, and perhaps most importantly, patients described it as a valuable resource [25]. The authors of this study highlighted that EC is most effective when used correctly, and offering counseling in an inviting and non-judgmental manner may optimize patient uptake of this education opportunity. 

It is also important to note the differences between states in pharmacist prescribing of routine hormonal contraception, as additional reproductive health training may also impact EC access and knowledge. Pharmacist-prescribed hormonal contraceptives were initially implemented in 2016, and there are currently eleven states which have implemented or are in the process of implementing this expanded scope of practice [16]. Although training differs from state to state, the typical requirement includes 1–4 h of continuing education specific to hormonal contraceptives, but some states also consider recent graduates adequately trained [3]. For example, California grants prescriptive authority for hormonal contraception to those who complete a doctorate of pharmacy from an accredited California college of pharmacy after 2014. California also maintains a separate protocol for pharmacist-prescribed EC in addition to hormonal contraception, which requires a minimum of 1 h of continuing education specific to EC. In contrast, many states, including Georgia, have not permitted pharmacists to prescribe any type of ongoing or emergency contraception, and therefore have not developed additional training or education in this area. 

Strengths of this study include the use of a standardized script, and inclusion of all retail community pharmacies that are open to the public within the cities evaluated. This data provides an analysis of the real-world counseling and stock information that is offered to patients when they seek EC. However, the study has limitations, as pharmacists may have had LNG or UPA in stock at the pharmacy, but the mystery caller did not inquire about stock unless the pharmacist mentioned the specific product. Therefore, this study may have underestimated the actual percentage of pharmacies that had EC in stock. Additionally, this study was conducted in 2017 and the number of states allowing pharmacist prescribing of hormonal contraception has increased since that time. Although hormonal contraception prescribing protocols typically do not include EC, broader pharmacist exposure to reproductive health topics could improve EC access and information from pharmacists in these states overall, and would not be captured in this data. This encounter was conducted over the telephone and interaction with the pharmacist may have been different if the patient had presented in person at the pharmacy. These three large central metro cities were selected as a convenience sample from two states, and the results may not be directly applicable to other states which have different patient populations. This data may not be applicable to rural areas, particularly because other studies have shown rural pharmacies may be even less likely to stock EC than their urban counterparts [26,27]. Lastly, California is often the first state to implement new health care innovations and public health strategies, including expanding the pharmacist scope of practice, which may create a different patient experience and therefore, limit the applicability of these results in other states. 

Additional research is needed to determine why pharmacists rarely discuss UPA with patients seeking EC, particularly since it is the most effective oral EC agent available. This study identifies gaps in pharmacist EC knowledge and practice, and suggests that pharmacists in states which do not permit prescribing of EC may be less likely to provide immediate access and accurate information regarding EC. Additional research should be conducted with pharmacists in these regions to determine how to best improve patient care and access to all EC methods.

## 5. Conclusions

Pharmacists practicing in states with expanded scopes allowing pharmacist-prescribed EC may be associated with providing improved access to oral EC and more accurate patient counseling. 

## Figures and Tables

**Table 1:** Pharmacy type and location.

Pharmacy Type	Total *n* = 388	CA *n* = 239	GA *n* = 149	*p* Value
Independent	78 (20.1%)	42 (17.6%)	36 (24.2%)	0.12
Chain-All	310 (79.9%)	197 (82.4%)	113 (75.8%)	
Chain-Drugstore	216 (55.7%)	147 (61.5%)	69 (46.3%)	--
Chain-Grocery	68 (17.5%)	32 (13.4%)	36 (24.2%)	--
Chain–Mass merchandiser	26 (6.7%)	18 (7.5%)	8 (5.4%)	--

**Table 2:** Pharmacist discussion and stock availability of oral emergency contraceptives by state.

EC Method Status	Total *n* = 388	CA *n* = 239	GA *n* = 149	*p* Value
LNG Discussed	337 (86.9%)	216 (90.4%)	121 (81.2%)	0.01
LNG Available	315 (81.2%)	214 (89.5%)	101 (67.8%)	<0.01
UPA Discussed	59 (15.2%)	54 (22.6%)	5 (3.4%)	<0.01
UPA Available	24 (6.2%)	23 (9.6%)	1 (0.7%)	<0.01

**Table 3:** Pharmacist discussion and stock availability of oral emergency contraceptives by pharmacy type.

EC Method Status	CA Chain *n* = 197	CA Independent *n* = 42	*p* Value	GA Chain *n* = 113	GA Independent *n* = 36	*p* Value
LNG Discussed	191 (97.0%)	25 (59.5%)	<0.01	106 (93.8%)	15 (41.7%)	<0.01
LNG Available	190 (96.4%)	24 (57.1%)	<0.01	92 (81.4%)	9 (25.0%)	<0.01
UPA Discussed	47 (23.9%)	7 (16.7%)	0.31	4 (3.5%)	1 (2.8%)	0.75
UPA Available	22 (11.2%)	1 (2.4%)	0.08	0	1 (2.8%)	0.54

**Table 4:** Pharmacist reported levonorgestrel emergency contraception (LNG EC) window of efficacy.

“It has been 4 days since I had sex, will it [LNG EC] still work?”	CA *n* = 166	GA *n* = 120	*p* Value
No	37 (22.3%)	90 (75.0%)	<0.01
Unsure	17 (10.2%)	9 (7.5%)	
Yes	15 (9.0%)	3 (2.5%)	
Yes but less effective	97 (58.4%)	18 (15.0%)	

**Table 5:** Unprompted pharmacist emergency contraception (EC) counseling points.

Pharmacist Counseling Points	CA *n* = 216	GA *n* = 121	*p* Value
Pharmacist mentions EC efficacy time frame	207 (95.8%)	112 (92.6%)	0.20
Asks when unprotected intercourse occurred	45 (20.8%)	14 (11.6%)	0.03
Asks patients age	5 (2.3%)	8 (6.6%)	0.05
Asks patients weight	1 (0.5%)	0 (0%)	0.45
Asks regarding sexually transmitted infection prevention	0	0	--
Counsels regarding ongoing contraception	4 (1.9%)	1 (0.8%)	0.46
Asks regarding patient insurance	6 (2.8%)	0 (0%)	0.06
Asks about prescription	38 (17.6%)	0 (0%)	<0.01
